# Supplementary material for: Hazardous thunderstorm intensification over Lake Victoria
Source: Nat Commun. 2016 Sep 23;7:12786. doi: 10.1038/ncomms12786 (PMC5036145; doi:10.1038/ncomms12786)
Supplement: Supplementary Information — Supplementary Figures 1-8, Supplementary Tables 1-2, Supplementary Notes 1-5 and Supplementary References [file ncomms12786-s1.pdf]

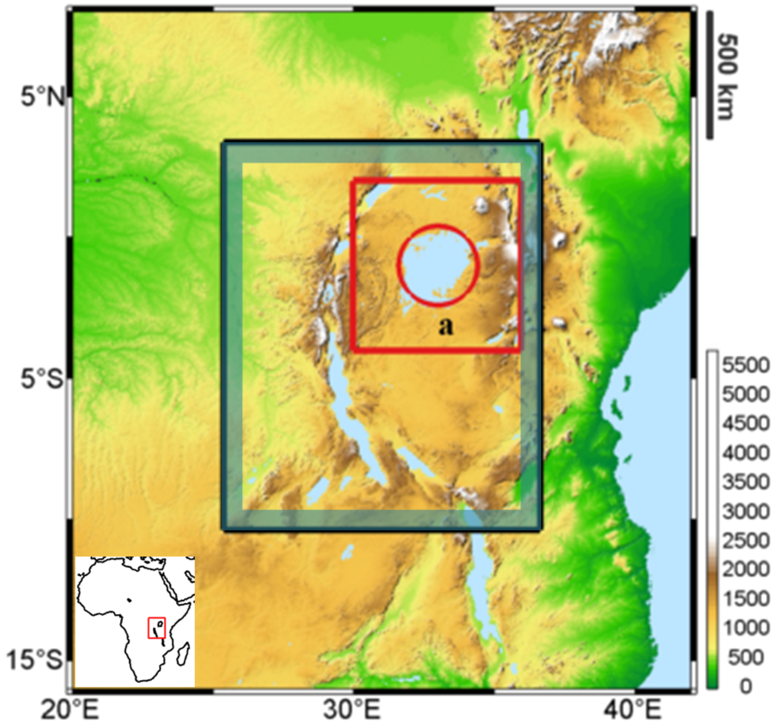

Supplementary Figure 1 | COSMO-CLM^2 domain description. Shuttle Radar Topography Mission (SRTM) surface elevation (m) over the African Great Lakes region. The black rectangle denotes the COSMO-CLM^2 high-resolution (0.0625°) model domain (25.375°E to 36.625°E; 10.375°S to 3.375°N, center at 31°E, 3.5°S) and the blue band a 10 grid point wide zone excluded from the analysis. Lake Victoria (a) is the largest of the African Great Lakes and the second largest freshwater lake in the world. For each pixel on the simplified Lake Victoria contour (red circle with 1.4° radius and centre at 1°S - 33°E), the 10 m wind vector is projected onto the radiant direction, after which vector length scalars are averaged along the circle yielding the lake breeze strength (outward defined positive). The same contour is also used in the Moisture Flux Convergence (MFC) framework. In this case the meridional or latitudinal wind component at the outer edge of each contour pixel is used to compute the MFC. The red rectangle finally includes the land pixels considered as "surrounding land" (30°E to 36°E; 4°S to 2°N).

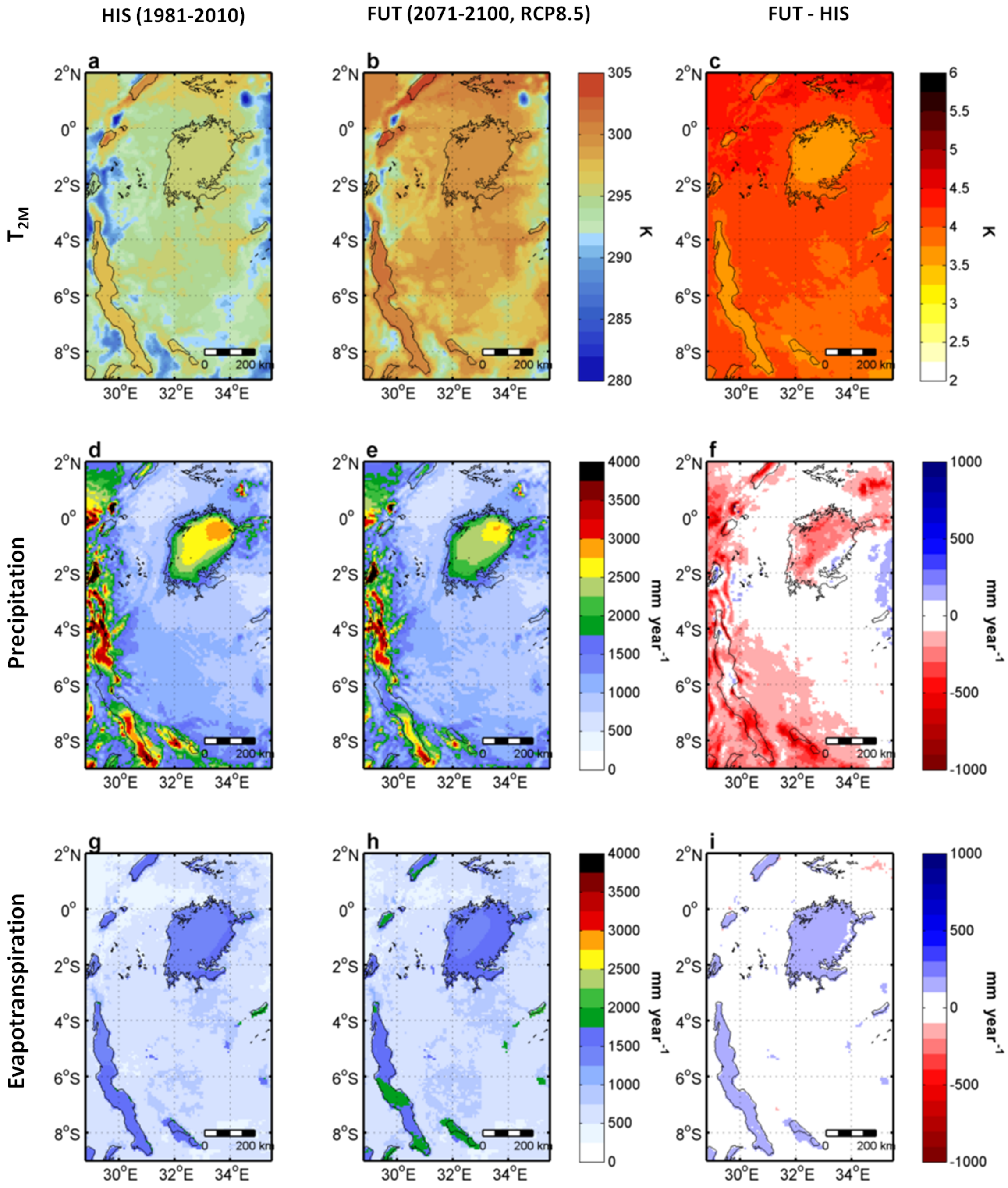

Supplementary Figure 2 | Projected climate change for the African Great Lakes region from COSMO-CLM^2. Shown are annual mean 2 m air temperature T_{2M} [K], precipitation [mm year^{-1}] and evapotranspiration [mm year^{-1}] from the **(a,d,g)** HIS (1981-2010) and **(b,e,h)** FUT (2071-2100) simulations, and **(c,f,i)** the change between both periods under RCP8.5.

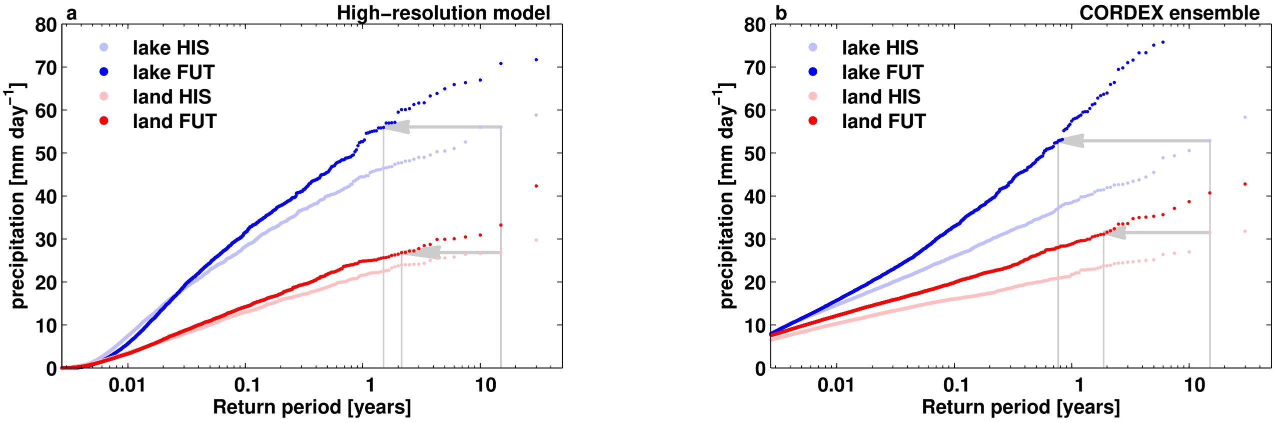

Supplementary Figure 3 | Projected end-of-century changes in precipitation return periods over Lake Victoria. a,b, 24h Lake (blue squares) and land (red squares) return periods from COSMO-CLM^2 and the ensemble of nine CORDEX-Africa members employing a lake model for their simulations, respectively. Light colors indicate historical (1981-2010) return periods while dark colors indicate projected (2071-2100) return periods under RCP8.5. Grey arrows indicate the change in return period for a 1-in-15 year event between the historical and future simulations.

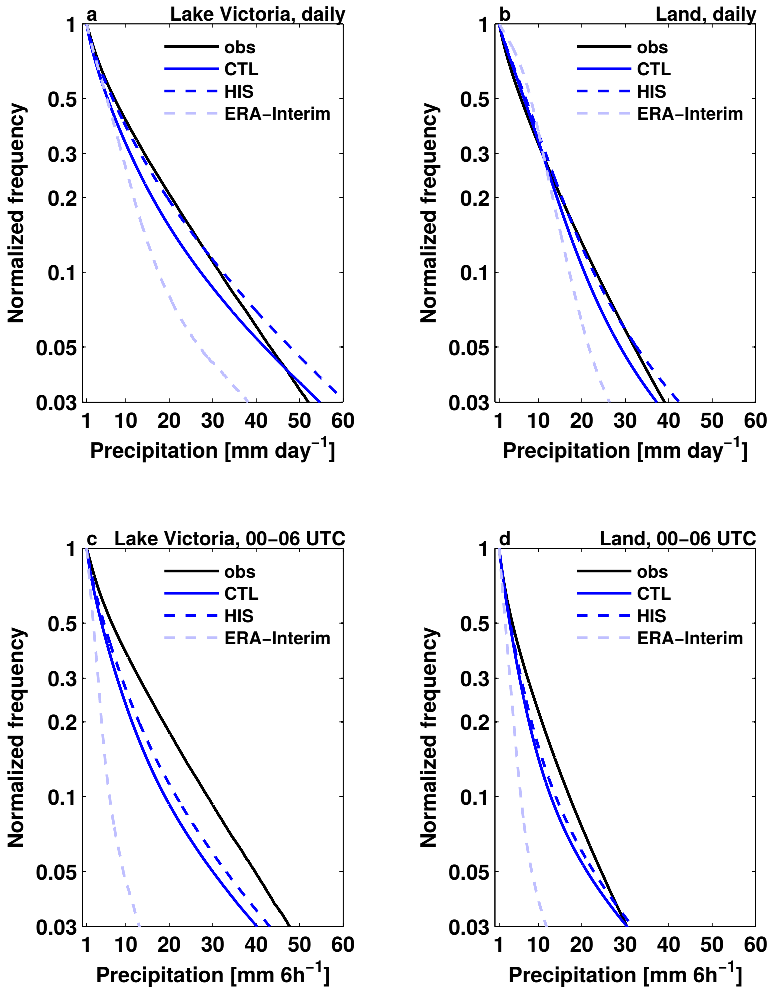

Supplementary Figure 4 | Evaluating COSMO-CLM^2 and ERA-Interim for precipitation distribution. Cumulative distributions of **a,b** daily and **c,d** 6-hourly precipitation during 1999-2008, expressed relative to the number of wet days (daily precipitation exceeding 1 mm d^{-1}), over Lake Victoria and all land area within the COSMO-CLM^2 model domain (Supplementary Fig. 1), respectively. ERA-Interim output was remapped to the COSMO-CLM^2 grid using nearest neighbour interpolation.

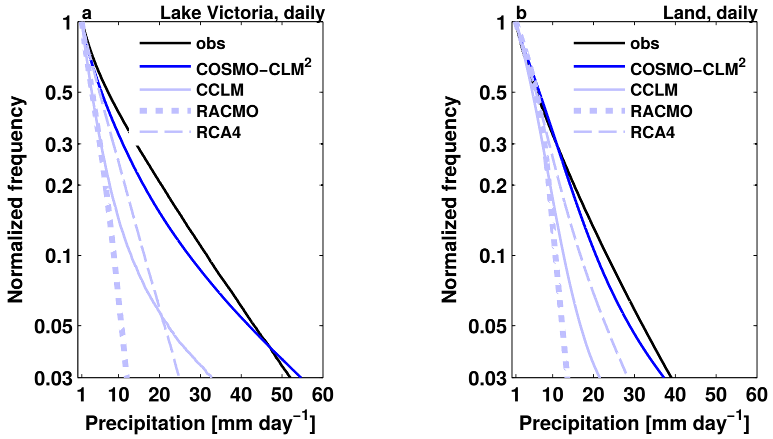

Supplementary Figure 5 | Evaluating COSMO-CLM^2 and CORDEX regional climate models (RCMs) for precipitation distribution. Same as Supplementary Fig. 4a,b, but instead of ERA-Interim showing the dynamical downscaling of ERA-Interim to 0.44° using three CORDEX RCMs (light blue lines): COSMO-CLM4.8 (CCLM), RACMO 22T (RACMO) and RCA4. The RCMs were selected on the public availability of CORDEX projections over Africa and their output was remapped to the COSMO-CLM^2 grid using nearest neighbour interpolation.

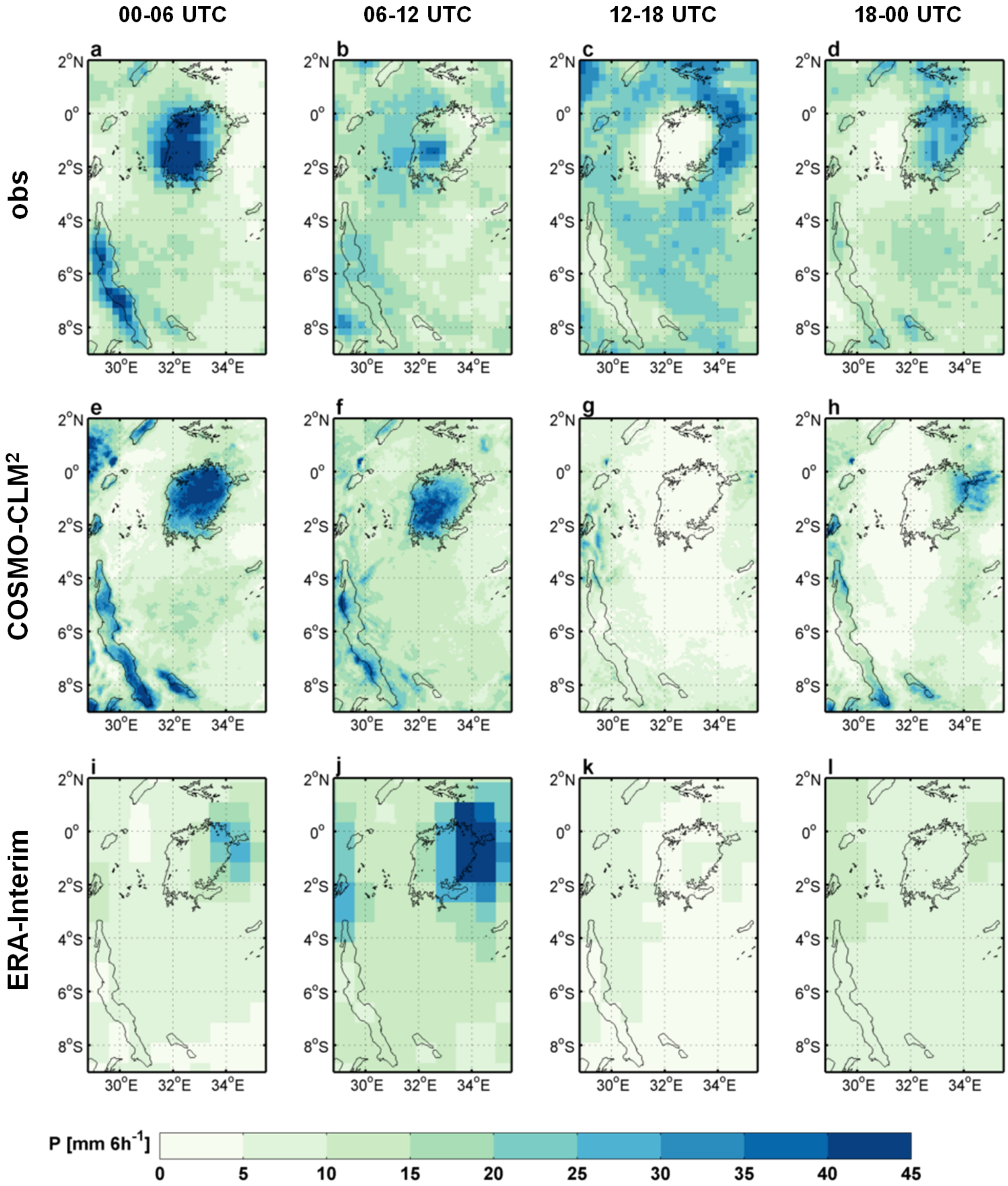

Supplementary Figure 6 | Evaluating COSMO-CLM^2 and ERA-Interim for extreme precipitation. 99% percentile maps of 6-hourly accumulated precipitation, as observed by TRMM (**a-d**) and as modelled by COSMO-CLM^2 (**e-h**) and ERA-Interim (**i-l**) during 1999-2008. TRMM and ERA-Interim output were remapped to the COSMO-CLM^2 grid using nearest neighbour interpolation.

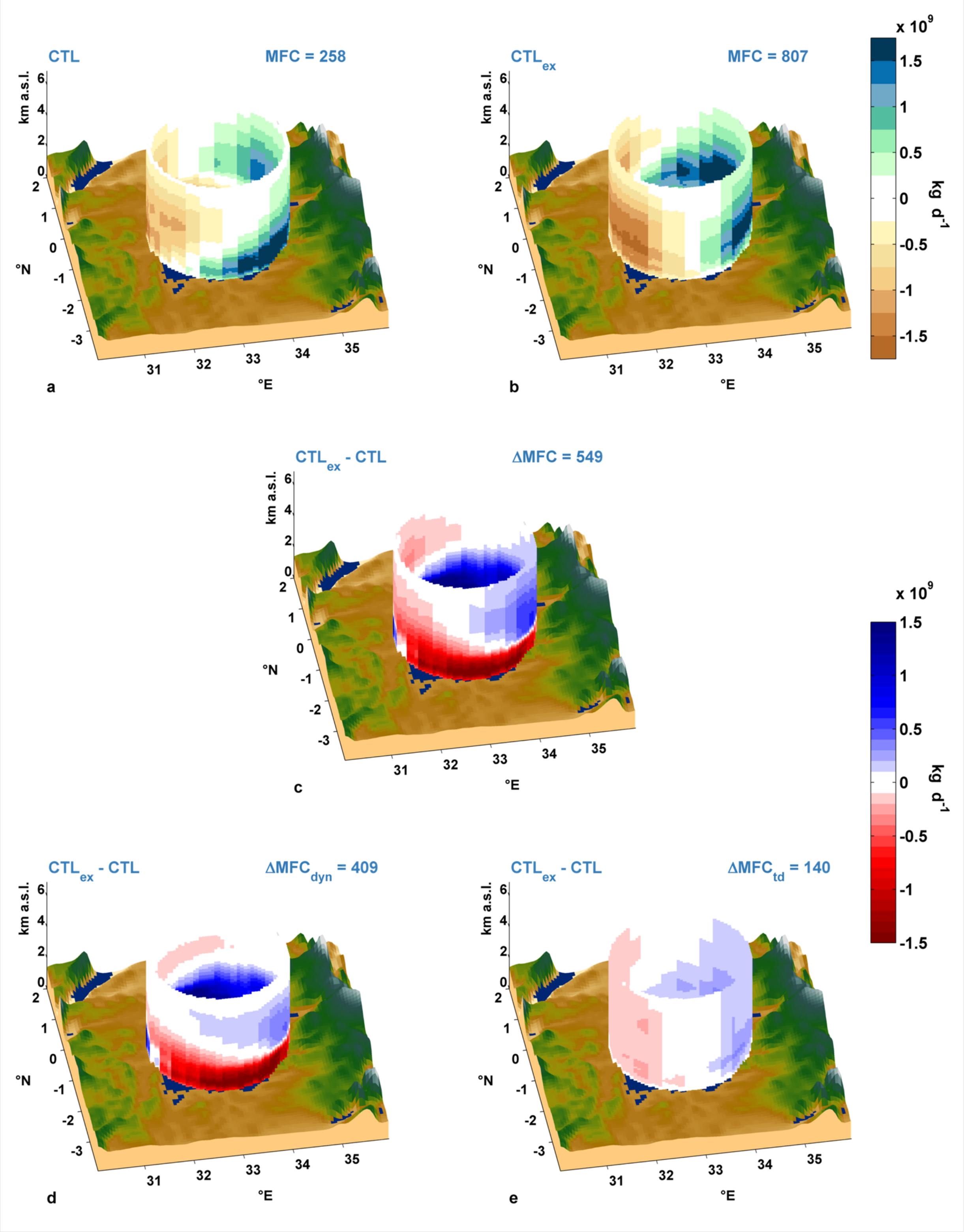

Supplementary Figure 7 | Illustrating the Moisture Flux Convergence (MFC) framework: Dynamic versus thermodynamic controls on present-day extremes. a, 1999-2008 daily accumulated MFC over Lake Victoria from the CTL simulation. **b**, Daily accumulated MFC from the CTL simulation for the 24h periods (09h-09h UTC) associated with nighttime precipitation above the 99th percentile (00h-09h UTC; CTL$_{ex}$). **c**, Daily accumulated MFC difference between CTL and CTL$_{ex}$. **d,e**, Daily accumulated MFC difference between CTL and CTL$_{ex}$ considering only dynamic and thermodynamic drivers, respectively. Spatially integrated MFC values are displayed in units 10^9 kg d^{-1}. See Methods for a description of the MFC computation and its separation into dynamic and thermodynamic contributions.

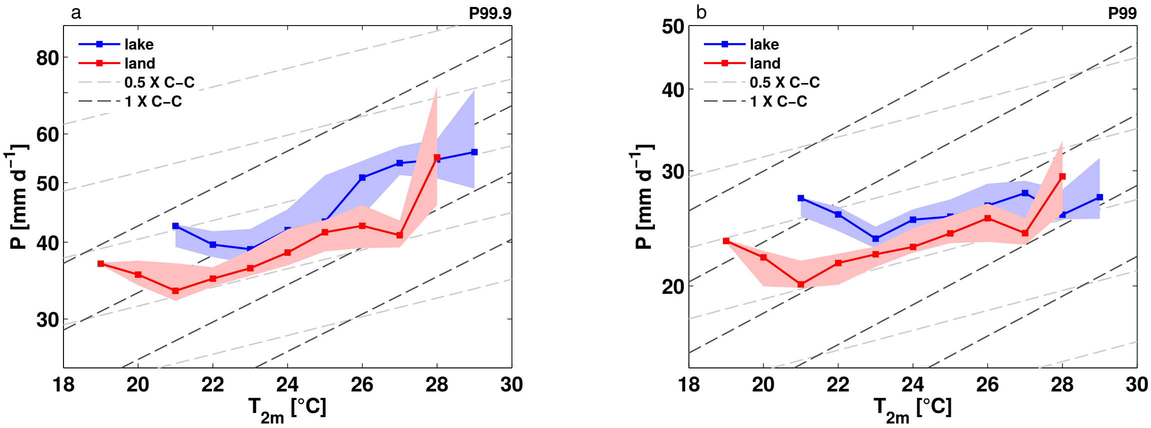

Supplementary Figure 8 | Testing Clausius-Clapeyron scaling. a, Blue and red lines indicate the ensemble median CORDEX daily 99.9th percentile precipitation intensity during 1950-2100 (only members using a lake model) as a function of temperature under RCP8.5 over Lake Victoria and surrounding land (red square in Supplementary Fig. 1), respectively. **b**, Same as **a**, but for the 99th percentile precipitation intensity. Coloured bands denote the interquartile range. Dashed lines are the exponential relations given by 0.5 (light grey) and 1 (dark grey) times the Clausius-Clapeyron relation (C-C). Note the logarithmic y-axis and different y-axis ranges.

Supplementary Table 1 | Overview of COSMO-CLM^2 and CORDEX model experiments. For each experiment the employed Regional Climate model (RCM), lateral boundary conditions (LBC), analysis period (excluding spin-up), horizontal grid resolution and lake parameterisation scheme (if any) is mentioned. COSMO-CLM^2 simulations start three years prior to the first analysis year. The CORDEX-EVAL simulations were only used for model evaluation purposes (Supplementary Fig. 5). CORDEX simulations 10-16 (highlighted in red) were omitted from the analysis since they were not coupled to a lake model. The FUT simulation was conducted for RCP8.5, whereas CORDEX simulations 1-16 are available both for RCP4.5 and RCP8.5 for the future period. The latter were used both for transient and time slice analyses (Clausius-Clapeyron scaling and comparison to COSMO-CLM^2, respectively).

Experiment name	RCM	LBC	Analysis period	Resolution	Lake model?
CTL	COSMO-CLM^2	ERA-Interim	1999-2008	0.0625°	FLake
NOL	COSMO-CLM^2	ERA-Interim	1999-2008	0.0625°	(no lakes)
HIS	COSMO-CLM^2	MPI-ESM-LR	1981-2010	0.0625°	FLake
FUT	COSMO-CLM^2	MPI-ESM-LR	2071-2100	0.0625°	FLake
CORDEX-EVAL 1	COSMO-CLM	ERA-Interim	1999-2008	0.44°	No
CORDEX-EVAL 2	RACMO	ERA-Interim	1999-2008	0.44°	No
CORDEX-EVAL 3	RCA4	ERA-Interim	1999-2008	0.44°	FLake
CORDEX 1	RCA4	CanESM2	1951-2100	0.44°	FLake
CORDEX 2	RCA4	CM5A-MR	1951-2100	0.44°	FLake
CORDEX 3	RCA4	CNRM-CM5	1951-2100	0.44°	FLake
CORDEX 4	RCA4	EC-EARTH	1951-2100	0.44°	FLake
CORDEX 5	RCA4	GFDL-ESM2M	1951-2100	0.44°	FLake
CORDEX 6	RCA4	HadGEM2-ES	1951-2100	0.44°	FLake
CORDEX 7	RCA4	MIROC5	1951-2100	0.44°	FLake
CORDEX 8	RCA4	MPI-ESM-LR	1951-2100	0.44°	FLake
CORDEX 9	RCA4	NorESM1-M	1951-2100	0.44°	FLake
CORDEX 10	COSMO-CLM	CNRM-CM5	1951-2100	0.44°	No
CORDEX 11	COSMO-CLM	EC-EARTH	1951-2100	0.44°	No
CORDEX 12	COSMO-CLM	HadGEM2-ES	1951-2100	0.44°	No
CORDEX 13	COSMO-CLM	MPI-ESM-LR	1951-2100	0.44°	No
CORDEX 14	RACMO	EC-EARTH	1951-2100	0.44°	No
CORDEX 15	HIRHAM5	EC-EARTH	1951-2100	0.44°	No
CORDEX 16	HIRHAM5	NorESM1-M	1951-2100	0.44°	No

Supplementary Table 2 | Dynamic and thermodynamic contributions to present-day extremes and projected changes in mean and extreme precipitation. Daily accumulated, total Moisture Flux Convergence change (ΔMFC_{tot}) over Lake Victoria between two model output samples, as well as the dynamical and thermodynamical contributions to the total change (ΔMFC_{dyn} and ΔMFC_{td}, respectively). Absolute MFC changes are displayed in units 10^9 kg d^{-1}. See Methods for a description of the MFC computation, its separation into dynamic and thermodynamic contributions and an overview of the different model experiments.

	ΔMFC_{tot}		ΔMFC_{dyn}		ΔMFC_{td}	
Experiment	abs	%	abs	%	abs	%
CTL_{ex} - CTL	+549	+213	+409	+159	+140	+55
FUT_{ex} - HIS_{ex}	+935	+27	-114	-3	+1049	+31

Supplementary Notes

Supplementary Note 1. Projected changes for the African Great Lakes region

This study presents the first high-resolution, dedicated climate projection for the African Great Lakes region (Supplementary Fig. 1). Under a high-emission scenario, near-surface air temperatures are projected to rise by +4.16 K on average over the model domain towards 2071-2100 (relative to 1981-2010), and by +3.74 K over the lake surfaces (Supplementary Fig. 2c). The projected temperature change is consistent with the ensemble mean 2-meter temperature projection of +4.0 K (+2.4 to +5.6 K) under RCP8.5 by the CMIP5 ensemble for the larger East Africa (11.3°S to 15°N, 25°E to 52°E).

Precipitation decreases by -8.0% on average over the model domain and by -7.5% over the lake surfaces. The average precipitation decline is for most part due to the decreases over the western part of the rift valley mountains and over the African Great Lakes (Supplementary Fig. 2f). The distinct decrease in precipitation over the African Great Lakes is consistent with the ensemble mean projection of 9 CORDEX members[1] that use a lake model and are currently available in the public domain. Precipitation projections between COSMO-CLM^2 and CORDEX generally agree also in terms of binned precipitation change, although the precipitation increase over the land surrounding Lake Victoria is more pronounced in the CORDEX ensemble (Fig. 2). Also the amplification effect on the intensification of extremes is consistent in COSMO-CLM^2 and the individual CORDEX members.

Changes in evapotranspiration are small over land (+31 mm yr^{-1} or +6%) but a relatively large absolute increase is projected over the lake surfaces where evaporation is already high (+142 mm yr^{-1} or +9%; Supplementary Fig. 2i). However, although high in absolute terms, this increase is insufficient to keep track with the enhanced water holding capacity of the warming troposphere.

Supplementary Note 2. Evaluation of COSMO-CLM^2 for extremes

The skill of the COSMO-CLM^2 model system has been assessed in several recent regional climate modelling studies[2–8]. In COSMO-CLM^2, the default land surface model of COSMO-CLM (TERRA-ML) is replaced by the Community Land Model version 3.5 (CLM3.5)[2,3]. The added value of COSMO-CLM^2 relative to the standard COSMO-CLM set-up was demonstrated both over Europe[2–4] and Central Africa[5], and arose from a better partitioning of the turbulent fluxes and associated improved representation of climate characteristics, such as near-surface temperature, precipitation and cloud cover. Based on the comparison of offline simulations with TERRA-ML and CLM3.5 (the land surface models of COSMO-CLM and COSMO-CLM^2, respectively) at four central African flux tower sites, this enhanced skill was attributed to a more realistic representation of the leaf area index, surface albedo and root depth in CLM3.5[5]. In a multiyear 25 km resolution COSMO-CLM^2 simulation over Central Africa, a close correspondence was obtained between observed and modeled near-surface temperature, column precipitable water and surface net longwave radiation, whereas the seasonal cycles of precipita-

tion, cloud cover, top of the atmosphere outgoing longwave radiation and various solar radiation quantities were mostly reproduced within the margins of observational uncertainty, albeit some regional deficiencies[6].

An extensive evaluation of the COSMO-CLM^2 CTL simulation over the African Great Lakes region (domain indicated on Supplementary Fig. 1) demonstrated the ability of the model system to represent the mean climate of the region considered in this study[8,9]. In particular, the resolution was deemed sufficient to capture effects of lakes and local orography on precipitation, wind and other atmospheric variables. Moreover, spatial and temporal patterns of the lake surface temperatures as well as differences between lakes were found to be well represented by the lake model embedded within COSMO-CLM^2 (FLake), confirming earlier findings from offline FLake simulations[10,11]. The mean annual cycles of net shortwave and longwave radiation at the surface, sensible and latent heat flux and cloud cover were also simulated mostly within the margins of observational uncertainty. A direct evaluation of the sensible and latent heat flux over the lake surfaces is so far not possible due to the lack of reliable observational reference, but the close reproduction of the lake surface temperature patterns suggest that the surface energy balance is reasonably well reproduced also over the African Great Lakes. Moreover, it was shown that the CTL simulation largely outperforms ERA-Interim for most of the considered variables, and the COSMO-CLM CORDEX-Africa evaluation simulation for precipitation, lake surface temperature and net surface shortwave radiation. Relative to these two other model products available for the African Great Lakes region, the CTL simulation benefits from the coupling to state-of-the-art land surface and lake models, as well as from the enhanced model resolution which allows for more fine-scale circulation and associated precipitation patterns to develop over this complex terrain.

Compared to evaluating the simulated mean climate over the African Great Lakes region, a model evaluation for precipitation extremes is more challenging. Since observational precipitation products are highly uncertain for extremes in the tropics[12], the reference products are to be treated with care and the evaluation is performed for 'moderate' extremes (e.g. 95th, 97.5^{th} or 99th percentile precipitation) rather than '(very) strong' extremes (e.g. 99.9^{th} or 99.99^{th} percentile precipitation). The percentiles are always shown against the number of wet days (defined here as days with precipitation > 1 mm), a well-established practice in climate science[13,14]. Although this approach may be misleading when studying precipitation changes (whereby the frequency of wet days may change itself and therewith incorrectly suggest changes in the computed percentiles[15]), it is appropriate to evaluate model output for the present-day climate[13]. Results reveal a reasonable agreement between TRMM and the COSMO-CLM^2 CTL simulation in terms of the frequency of daily precipitation events over Lake Victoria and the surrounding land (Supplementary Fig. 4a-b). In both cases, precipitation modelled by COSMO-CLM^2 represents a major improvement relative to the simulation of precipitation in ERA-Interim, as well as relative to three regional climate model simulations from the CORDEX-Africa initiative (Supplementary Fig. 4, 5). Also on subdaily time scales, TRMM and COSMO-CLM^2 agree rather well and the added value of COSMO-CLM^2 compared to ERA-Interim is manifest (ex-

emplified here by the nighttime precipitation, Supplementary Fig. 4c-d). Finally, changing the boundary conditions from ERA-Interim (CTL) to a free-running Global Climate Model (HIS) has only a small influence on COSMO-CLM^2's ability to reproduce the observed precipitation distribution (Supplementary Fig. 4), suggesting that this change in boundary conditions is no large source of bias within the domain.

COSMO-CLM^2 captures the strong lake imprint on nighttime extreme precipitation occurrence as well as the development of extremes through space and time (Supplementary Fig. 6). In particular, the cycle of convective initiation in the northeast lake sector from 18h-00h UTC (Supplementary Fig. 6j-k), followed by intensification and advection in westerly direction along the synoptic flow from 00h-06h UTC (Supplementary Fig. 6a-b), and the phasing out with reduced extreme precipitation values towards the western shore from 06h-12h UTC (Supplementary Fig. 6d-e). Again, ERA-Interim fails to reproduce the observed patterns, confirming the added value of COSMO-CLM^2, in particular through its dynamical downscaling of the reanalysis to high resolution and the inclusion of a lake model. During daytime, COSMO-CLM^2 underestimates convective precipitation over land, especially east of Lake Victoria where interactions between the synoptic and mesoscale circulation generate enhanced thunderstorm activity (Supplementary Fig. 6g-h). It is well known that convection parameterisations in regional climate models induce a number of biases in the representation of precipitation, including for instance errors in the diurnal cycle or the underestimation of heavy precipitation[13,15–18]. The absence of the afternoon precipitation maximum East of Lake Victoria moreover suggests another problem related to convection parameterisation. Using a set of convection-permitting simulations, Lauwaet et al. (2012)[19] showed how the cold pool of convective storms interacts with the cold pool of the Lake Chad to generate precipitation upwind of the lake. Such interactions occurring over Lake Victoria are not captured by the CTL simulation. We therefore assume that higher resolution simulations with explicitly resolved convection will lead to an improved representation of precipitation especially over land surrounding Lake Victoria.

Supplementary Note 3. Correlation versus causality

Application of the binning procedure to observations and the CTL simulation highlights a strong relationship between afternoon land precipitation and nighttime lake precipitation over Lake Victoria (Fig. 3). By itself this relationship does not imply causality, in fact, there may be a third factor controlling both land and lake precipitation (e.g. synoptic-scale moisture transport). Comparison of the CTL simulation to a simulation whereby all lake pixels have been replaced by representative land (NOL) allows us to separate the influence of Lake Victoria from all other effects, including synoptic-scale moisture transport[8]. Results of this comparison indicate that the mesoscale lake effect attributes for 74% of the median nighttime lake precipitation in each bin, 43% in the bins above the 90th percentile and 34% in the bins above the 99th percentile. From this analysis we conclude that large-scale processes alone cannot explain the occurrence of extremes over Lake Victoria, and that mesoscale lake effects are a positive feedback enhancing nighttime thunderstorm intensity.

Our results also highlighted the importance of changes in local atmospheric moisture content and mesoscale processes for understanding the future intensification of precipitation extremes over Lake Victoria. However, it is possible that large-scale variability and climate change modulate these mesoscale interactions. Given the limited domain of our high-resolution model, this question cannot be fully addressed in the present study. Further research is required to fully understand large scale drivers of local precipitation variability and change in the Lake Victoria basin. This could, for instance, be achieved through surrogate climate scenarios whereby thermodynamic changes play but atmospheric dynamics are not affected[20,21].

Supplementary Note 4. Moisture Flux Convergence

By applying the Moisture Flux Convergence framework (MFC) to the COSMO-CLM^2 output, it is possible to quantify the moisture convergence over Lake Victoria for a given time period (Methods). Supplementary Fig. 7a shows the 24h average moisture fluxes in the CTL simulation. Spatial integration yields an average MFC of 258 x 10^9 kg d^{-1} from 1999 to 2008. While the atmospheric column loses moisture towards the west, the moisture gain from the east is larger, resulting in a net convergence of moisture. During 24h periods associated with extreme nighttime precipitation (Supplementary Fig. 7b), MFC more than triples to 807 x 10^9 kg d^{-1}. The total change (Supplementary Fig. 7c) can subsequently be split up into a dynamic (Supplementary Fig. 7d) and thermodynamic contribution (Supplementary Fig. 7d; see Methods how this is done). Results show that the dynamic component is the dominant contributor (74%) to the increase in MFC during extremes (Supplementary Table 2). Note that the relative contribution to the precipitation change may depend on the considered percentile. Investigating this sensitivity is however beyond the scope of the present study.

The same procedure was applied to quantify the future change in MFC during extremes only (Supplementary Table 2). In this case, the increase in MFC by +27% is entirely due to the thermodynamic component, that is, a higher moisture content of advected air masses instead of mesoscale circulation changes.

Supplementary Note 5. Clausius-Clapeyron scaling

Many studies have related the increase of precipitation extremes with global warming to the Clausius-Clapeyron relation[12,15,22–24], which expresses the increase of the water-holding capacity of the atmosphere with warmer temperatures and sets the sensitivity to 6-7% K^{-1} for typical surface temperatures[25]. Others have suggested that subdaily precipitation extremes may exceed expectations from the Clausius-Clapeyron relation (super-Clausius-Clapeyron scaling) based on the analysis of rain gauge data[13,26–28] and historical[13,26] and future[14,26] climate simulations. Here we assess the expected Clausius-Clapeyron scaling for daily, very extreme precipitation (exceeding the 99.9^{th} percentile - that is, expected once in about three years) over this large tropical lake.

To start, we compute the 99.9^{th} percentile precipitation per CORDEX simulation and per

decade during 1950-2100. The arithmetic averages over Lake Victoria and surrounding land (4.01°S - 2.01°N and 29.98°E - 35.95°E) are subsequently binned according to the corresponding decadal mean 2-meter air temperatures over lake and land using a bin width of 1 K. Medians, first and third quartiles are computed for each bin and visualised in Supplementary Fig. 8. Clausius-Clapeyron scaling factors over the whole 150-year simulation period are obtained per simulation through a linear fit to the natural logarithm of the spatially averaged decadal mean 99.9^{th} percentiles of daily lake and land precipitation, respectively. The same approach is applied to compute the Clausius-Clapeyron scaling factors from the COSMO-CLM^2 HIS (1981-2010) and FUT (2071-2100) simulations, and to test the sensitivity of the scaling to the precipitation percentile and choice of the RCP.

For the selected CORDEX members (1950-2100), the ensemble mean scaling for the 99.9^{th} daily precipitation percentile is 7.9% K^{-1} over Lake Victoria and substantially higher compared to the 5.5% K^{-1} sensitivity over surrounding land (Supplementary Fig. 8a; the ensemble median is 8.0% K^{-1} over the lake and 4.7% K^{-1} over land). In the high-resolution COSMO-CLM^2 output (1981-2010 and 2071-2100), 99.9^{th} daily precipitation extremes scale by 6.5% K^{-1} over Lake Victoria and 3.7% K^{-1} over the surrounding land, corresponding to about 1 and 0.6 times the Clausius-Clapeyron scaling, respectively. Although sensitive to the choice of the RCP and extreme precipitation index (Supplementary Fig. 8b), super-Clausius-Clapeyron scaling is found in none of the cases, and over land the scaling remains below 1 time Clausius-Clapeyron even for the strongest extremes.

The distinct scaling over lake and land for very extreme precipitation is remarkable, considering the fact that scaling patterns in the extra-tropics generally display little geographical structure, even in the presence of strong orography[15]. It highlights the key role of Lake Victoria as a regulator of future extremes. Only over the lake, the future moisture content of converging air masses will be high enough to sustain the thermodynamic rate. Over land, moisture availability constrains future extreme precipitation increases expected from thermodynamic considerations. This is in agreement with an analysis of the CMIP5 projections for the region suggesting an increased deficit in local moisture supply[29] and associated increased dependence of regional precipitation to moisture influx from the Indian Ocean[30]. In addition, it has already been suggested that the thermodynamic constraint may not be a good predictor in tropical regions experiencing circulation changes[31]. In previous studies, changes in the moist-adiabatic temperature lapse rate, tropospheric temperatures and vertical velocity during extremes[32] have been advanced next to increased atmospheric moisture content[26] as explanations for more intense precipitation extremes[31]. However, while the thermodynamic argument for extreme precipitation sensitivity to temperature changes is well known, other, dynamical factors have been much less studied[25].

Supplementary References

[1] Giorgi, F., Jones, C. & Asrar, G. Addressing climate information needs at the regional level: the CORDEX framework. *WMO Bulletin* **58**, 175–183 (2009).

[2] Davin, E. L., Stöckli, R., Jaeger, E. B., Levis, S. & Seneviratne, S. I. COSMO-CLM2: a new version of the COSMO-CLM model coupled to the Community Land Model. *Climate Dynamics* **37**, 1889–1907 (2011).

[3] Davin, E. L. & Seneviratne, S. I. Role of land surface processes and diffuse/direct radiation partitioning in simulating the European climate. *Biogeosciences* **9**, 1695–1707 (2012).

[4] Lorenz, R., Davin, E. L. & Seneviratne, S. I. Modeling land-climate coupling in Europe: Impact of land surface representation on climate variability and extremes. *Journal of Geophysical Research* **117**, D20109 (2012).

[5] Akkermans, T. *et al.* Validation and comparison of two soil-vegetation-atmosphere transfer models for tropical Africa. *Journal of Geophysical Research* **117**, G02013 (2012).

[6] Akkermans, T., Thiery, W. & Van Lipzig, N. P. M. The Regional Climate Impact of a Realistic Future Deforestation Scenario in the Congo Basin. *Journal of Climate* **27**, 2714–2734 (2014).

[7] Lejeune, Q., Davin, E. L., Guillod, B. P. & Seneviratne, S. I. Influence of Amazonian deforestation on the future evolution of regional surface fluxes, circulation, surface temperature and precipitation. *Climate Dynamics* **44**, 2769–2786 (2015).

[8] Thiery, W. *et al.* The Impact of the African Great Lakes on the Regional Climate. *Journal of Climate* **28**, 4061–4085 (2015).

[9] Docquier, D., Thiery, W., Lhermitte, S. & van Lipzig, N. P. M. Multi-year wind dynamics around Lake Tanganyika. *Climate Dynamics* (2016).

[10] Thiery, W. *et al.* Understanding the performance of the FLake model over two African Great Lakes. *Geoscientific Model Development* **7**, 317–337 (2014).

[11] Thiery, W. *et al.* LakeMIP Kivu: Evaluating the representation of a large, deep tropical lake by a set of 1-dimensional lake models. *Tellus A* **66**, 21390 (2014).

[12] Kharin, V. V., Zwiers, F. W., Zhang, X. & Wehner, M. Changes in temperature and precipitation extremes in the CMIP5 ensemble. *Climatic Change* **119**, 345–357 (2013).

[13] Ban, N., Schmidli, J. & Schär, C. Evaluation of the convection-resolving regional climate modeling approach in decade-long simulations. *Journal of Geophysical Research: Atmospheres* **119**, 7889–7907 (2014).

[14] Kendon, E. *et al.* Heavier summer downpours with climate change revealed by weather forecast resolution model. *Nature Climate Change* **4**, 570–576 (2014).

[15] Ban, N., Schmidli, J. & Schär, C. Heavy precipitation in a changing climate : Does short-term summer precipitation increase faster? *Geophysical Research Letters* **42**, 1165–1172 (2015).

[16] Kendon, E. J., Roberts, N. M., Senior, C. A. & Roberts, M. J. Realism of rainfall in a very high-resolution regional climate model. *Journal of Climate* **25**, 5791–5806 (2012).

[17] Prein, A. F. *et al.* A review on regional convection-permitting climate modeling: Demonstrations, prospects, and challenges. *Reviews of Geophysics* **53**, 323–361 (2015).

[18] Brisson, E. *et al.* How well can a convection-permitting climate model reproduce decadal statistics of precipitation, temperature and cloud characteristics? *Climate Dynamics* (2016).

[19] Lauwaet, D., van Lipzig, N. P. M., Van Weverberg, K., De Ridder, K. & Goyens, C. The precipitation response to the desiccation of Lake Chad. *Quarterly Journal of the Royal Meteorological Society* **138**, 707–719 (2012).

[20] Schär, C., Frei, C., Lüthi, D. & Davies, H. C. Surrogate climate-change scenarios for regional climate models. *Geophysical Research Letters* **23**, 669–672 (1996).

[21] van Lipzig, N. P. M., van Meijgaard, E. & Oerlemans, J. Temperature sensitivity of the Antarctic surface mass balance in a regional atmospheric climate model. *Journal of Climate* **15**, 2758–2774 (2002).

[22] Allen, M. R. & Ingram, W. J. Constraints on future changes in climate and the hydrologic cycle. *Nature* **419**, 224–232 (2002).

[23] O'Gorman, P. A. Sensitivity of tropical precipitation extremes to climate change. *Nature Geoscience* **5**, 697–700 (2012).

[24] Chan, S. C., Kendon, E. J., Roberts, N. M., Fowler, H. J. & Blenkinsop, S. Downturn in scaling of UK extreme rainfall with temperature for future hottest days. *Nature Geoscience* **5**, 1–6 (2015).

[25] O'Gorman, P. A. Precipitation Extremes Under Climate Change. *Current Climate Change Reports* **1**, 49–59 (2015).

[26] Lenderink, G. & van Meijgaard, E. Increase in hourly precipitation extremes beyond expectations from temperature changes. *Nature Geoscience* **1**, 511–514 (2008).

[27] Berg, P., Moseley, C. & Haerter, J. O. Strong increase in convective precipitation in response to higher temperatures. *Nature Geoscience* **6**, 181–185 (2013).

[28] Loriaux, J. M., Lenderink, G., De Roode, S. R. & Siebesma, P. A. Understanding Convective Extreme Precipitation Scaling Using Observations and an Entraining Plume Model. *Journal of Atmospherical Science* **70**, 3641–3655 (2013).

[29] Otieno, V. O. & Anyah, R. O. CMIP5 simulated climate conditions of the Greater Horn of Africa (GHA). Part II: projected climate. *Climate Dynamics* **41**, 2099–2113 (2013).

[30] Shongwe, M., van Oldenborgh, G. J., van der Hurk, B. & van Aalst, M. Projected Changes in Mean and Extreme Precipitation in Africa under Global Warming. Part II: East Africa. *Journal of Climate* **24**, 3718–3733 (2011).

[31] Seneviratne, S. I. *et al.* Changes in climate extremes and their impacts on the natural physical environment. In Field, C. *et al.* (eds.) *Managing the Risks of Extreme Events and Disasters to Advance Climate Change Adaptation*, 109–230 (Cambridge University Press, Cambridge, UK, 2012).

[32] O'Gorman, P. A. & Schneider, T. The physical basis for increases in precipitation extremes in simulations of 21st-century climate change. *Proceedings of the National Academy of Sciences of the United States of America* **106**, 14773–14777 (2009).
